# Exploring positive pathways to care for members of the UK Armed Forces receiving treatment for PTSD: a qualitative study

**DOI:** 10.3402/ejpt.v5.21759

**Published:** 2014-02-17

**Authors:** Dominic Murphy, Elizabeth Hunt, Olga Luzon, Neil Greenberg

**Affiliations:** 1 King’s Centre for Military Health Research, King’s College London, London, UK; 2 Department of Clinical Psychology, Royal Holloway University, London, UK

**Keywords:** Military health, PTSD, depression, pathways, stigma, barriers

## Abstract

**Objective:**

To examine the factors which facilitate UK military personnel with post-traumatic stress disorder (PTSD) to engage in help-seeking behaviours.

**Methods:**

The study recruited active service personnel who were attending mental health services, employed a qualitative design, used semi-structured interview schedules to collect data, and explored these data using interpretative phenomenological analysis (IPA).

**Results:**

Five themes emerged about how participants were able to access help; having to reach a crisis point before accepting the need for help, overcoming feelings of shame, the importance of having an internal locus of control, finding a psychological explanation for their symptoms and having strong social support.

**Conclusions:**

This study reported that for military personnel who accessed mental health services, there were a number of factors that supported them to do so. In particular, factors that combated internal stigma, such as being supported to develop an internal locus of control, appeared to be critical in supporting military personnel to engage in help-seeking behaviour.

Since 2002, the UK and US military’s have conducted highly challenging operations in Afghanistan and Iraq. These military operations have been the focus of a number of large-scale epidemiological research studies, which have investigated the psychological health of US and UK service personnel. Studies in the United States have observed rates of post-traumatic stress disorder (PTSD) in deployed personnel to be between 8 and 18% (Hoge et al., 2004; Smith et al., 2008). Further, 13% of participants met criteria for alcohol problems and 18% for symptoms of anxiety and depression, with a very high co-morbidity rate between these disorders and PTSD (Riddle et al., 2007; Smith et al., 2008). This increase in the rate of PTSD following deployment has been replicated prospectively (Vasterling et al., 2006). However, in the UK, the effects of the conflict upon the mental health of service personnel have been quite different.

The most extensive UK epidemiological studies of service personnel since 2003 have been carried out at King’s College London. This study is based on a randomly selected representative sample of the UK military, and in 2006, this study reported rates of PTSD to be 4% and symptoms of common mental health problems (including anxiety and depression) to be 20% (Hotopf et al., 2006); higher rates of PTSD (6%) were found in combat troops and reserve forces. These rates remained reasonably constant at the second wave of data collection in 2010 (Fear et al., 2010). However, figures released by the Ministry of Defence (MoD) demonstrate substantially lower rates of personnel accessing services for these problems, between 4–4.5% and 0.8–1.2%, respectively, over the past 3 years (Defence Analytical Services Agency, 2011). This is supported by research that reported that only 23% of UK service personnel who meet criteria for a mental health diagnosis are receiving any support from mental health services (Iversen et al., 2010). Of those who engaged in help-seeking, 77% were getting treatment, with 56% receiving medication, 51% psychological therapy and 3% inpatient treatment.

A study within the UK Armed Forces followed up service personnel who had been involved in a 6-year longitudinal study, 3 years later (Iversen et al., 2005b). The study observed that most ex-service personnel do well once they leave. However, those who had a mental health problem when they left the Armed Forces were substantially more likely to be suffering from a mental health problem and be unemployed 3 years after leaving (Iversen et al., 2005b). In addition, having a mental health problem predicted leaving the Armed Forces and mental health status remained constant after leaving (Iversen et al., 2005b).

As documented above, only a modest number of military personnel experiencing mental health difficulties are able to access treatment, and little is known about the treatment experiences of military personnel who do access services (Iversen et al., 2009). What we do know is that many ex-service personnel are able to get treatment from the NHS, which provides a range of specialist services. Previous research has identified a number of barriers that may explain the reluctance to access services (Britt, Wright, & Moore, 2012; Gould et al., 2010; Iversen et al., 2011; Kim, Thomas, Wilk, Castro, & Hoge, 2010). These barriers broadly fit within three categories: internal stigma (including self-stigma), external stigma (including public stigma and mistrust in services), and access factors (including lack of knowledge of available services). Several trials have been conducted to improve the number of people seeking treatment by aiming to reduce stigma. A review of these trials concluded that there has been little evidence of the efficacy of these interventions (Mulligan, Fear, Jones, Wessely, & Greenberg, 2011).

The current study aims to investigate the specific pathways to accessing mental health services for members of the UK Armed Forces. In particular, to elucidate factors that support individuals to access services, and where barriers exist, how these are overcome. This is in line with the agenda of military occupational mental health services that have prioritised the importance of supporting individuals to access services at the earliest opportunity.

## Methods

### Setting & design

This study utilised a sample of UK service personnel who are accessing defence mental health services. Two military departments of community mental health (DCMHs) located in the south east of England were selected as they were geographically close to the investigating team; DCMHs provide services to all military personnel. The MoD and RHUL ethics committees granted ethical approval for this study.

A qualitative methodology was adopted for this study due to the exploratory nature of the research questions under investigation. The aim of the research questions was to understand the lived experiences of participants during their pathways to accessing mental health services, and interpretative phenomenological analysis (IPA) has been argued to be the most appropriate qualitative analytic approach to do this (Smith, Flowers, & Larkin, 2009).

### Participants

A sample size of between 8 and 10 participants was decided upon as informed by the selection of IPA (Smith & Osborn, 2008). An *ad hoc* sampling strategy was used for this study. The lead author (D. M.) met clinicians at the DCMHs and explained the inclusion and exclusion criteria. Clinicians were then requested to ask the clients who met these criteria whether they wished to participate in the study. Inclusion criteria for selection into the study included having a diagnosis of either PTSD or depression and currently receiving treatment. Individuals were not selected if they were in the process of being medically discharged from the military due to disciplinary reasons (this exclusion criteria was requested by the MoD ethics committee and the authors do not have access to the reasons why service personnel were being discharged), or if there was a clinical reason that meant it would not be appropriate for the individual to take part in the study. In general, these clinical reasons were if clients were new to the service. Clinicians were concerned that the study may be seen as an additional source of stress at a time when clients were first engaging in treatment and could have potentially created a barrier to their engagement in treatment.

### Materials

A semi-structured interview schedule was used. Broadly, the aim of the interview schedule was to understand the different pathways that participants’ took to access services, including which factors enabled them to do so, and how they overcame potential internal and external barriers. The interview schedule was piloted with three individuals who were accessing defence mental health services. The aim of this was to ensure that the questions were understandable and to check whether additional questions needed to be added. Following this, the interview schedule was refined taking into account feedback from a number of pilot interviews. This included advice about removing a number of questions and clarifying the stems of several questions.

Participants were also asked to complete two measures to record symptoms of mental illness. The Post Traumatic Checklist (PCL-C) is a self-report 17-item measure of the 17 DSM-IV symptoms of PTSD (Weather & Ford, 1996). The PCL-C has been previously validated against a clinical interview, which recommended using a cut-off of 50 or more (Blanchard, Jones-Alexander, Buckley, & Forneris, 1996). The Patient Health Questionnaire (PHQ-9) is a self-report measure that is based directly upon the DSM-IV criteria for depression and includes nine items. The PHQ-9 is scored from 0 to 27, and scores give an indication of symptom severity; scores between 15 and 19 indicates moderate to severe depression and a score of 20 or above indicates major depression (Kroenke & Spitzer, 2002). Participants were also asked a number of questions about their demographic characteristics.

### Procedure

Recruitment was carried out between March 2012 and June 2012. The DCMH staff were approached, and the inclusion and exclusion criteria for the study were discussed and a list of potential participants was drawn up. After initial consent had been granted for their details to be passed on from their treating clinician, potential participants were contacted to discuss the study, seek consent for them to be recruited, and find a suitable date and time to conduct the interview.

### Analysis

The first stage of data analysis was to collate the demographic characteristics and data collected through the standardised measures (PCL-C and PHQ-9). The second stage involved analysing the qualitative data in accordance with published guidelines for conducting IPA (Smith & Osborn, 2008; Willig, 2008). In brief, this involved working through a number of different stages. The first stage was to become familiar with the first participant’s transcript. The second stage was to make initial notations for ideas and themes in the text. The notations remained close to the participant’s words. The third stage was to develop emerging themes by re-reading the initial notations and assigning labels. The aim of these labels was to capture the essence of what the participant had described. The fourth stage was to search for connections between emerging themes. The list of labels was scrutinised and emergent themes that appeared to be connected to each other were grouped together under super-ordinate themes. Super-ordinate themes were broader in scope than emergent themes and contained a number of associated sub-themes. This process was then repeated for the next participant’s transcript. Once analysis had been completed for each transcript, a final master list of super-ordinate and sub-themes was generated. During this stage, differences and similarities between cases were noted. At this stage, themes between transcripts were grouped together and re-labelled where appropriate.

## Results

### Sample

Recruitment was carried out at two DCMHs. The sample consisted of 8 participants, with four from each DCMH. For the purposes of the study, participants were assigned *pseudonym*s to protect their anonymity.

Data were collected on participants’ socio-demographic characteristics to situate the sample; these are described in Table 1. The majority of the sample were male (six out of eight), in a relationship (7/8), had children (6/8), were Other Ranks and not officers (5/8), were British (7/8) and reported their ethnicity to be white (8/8). The ages of participants ranged from early 20s to mid-50s, with the majority or participants aged between mid-20s and mid-30s. The lengths of service varied from 4 to 31 years, with the mean length of service approximately 13 years. Nearly, 50% of the sample was in the Royal Navy and 50% was in the Army.

**Table 1:** Socio-demographic characteristics of the sample

Participant	Sex	Age	Relationship status	Children	Nationality	Ethnicity	Service	Rank (officer or in ranks)	Years in military
P1	Male	42	Divorced	Yes	British	White	Army	Officer	23
P2	Male	51	Married	Yes	British	White	Navy	Officer	31
P3	Male	34	Married	Yes	British	White	Navy	Officer	14
P4	Male	30	Married	Yes	British	White	Navy	Ranks	11
P5	Female	27	Partner	No	British	White	Navy	Ranks	10
P6	Female	22	Partner	No	British	White	Army	Ranks	4
P7	Male	31	Married	Yes	British	White	Army	Ranks	4
P8	Male	35	Married	Yes	New Zealand	White	Army	Ranks	6

Rates of mental health are reported in Table 2. The results indicate that three of the participants reported clinically significant levels of distress at the time of the interview, as measured on both the PHQ-9 and PCL-C. In addition, two further participants’ scores approached the cut-offs that defined case criteria on both of the measures. One of the inclusion criteria for the study was that participants had a diagnosis of PTSD or major depression. The observed variation in rates of distress may be indicative of participants being at different stages of treatment at the time the interviews were conducted.

**Table 2:** PHQ-9 and PCL-C scores for sample

Participant	PHQ-9 score^1^	Met criteria for PHQ-9 case	PCL-C score^2^	Met criteria for PCL-C case
P1	13	No	41	No
P2	4	No	8	No
P3	0	No	8	No
P4	23	Major depression	80	Yes
P5	4	No	28	No
P6	12	No	40	No
P7	21	Major depression	71	Yes
P8	17	Moderate to severe depression	63	Yes

1 PHQ-9 scored from 0 to 27: scores 15–19 indicates moderate to severe depression and a score of 20 or above indicates major depression

2 PCL-C scored from 17 to 85; scores above 50 indicates meeting criteria for post-traumatic stress reactions.

### Results of qualitative analysis

Five super-ordinate themes emerged from the data. Each of these super-ordinate themes contained a number of sub-themes; these are presented in Table 3.

**Table 3:** Master list of super-ordinate and sub-themes

Super-ordinate themes	Sub-themes
Recognising something was wrong	Reaching a crisis point
	Difficulties experienced as physical symptoms
Overcoming internal stigma	Shame
	Stigma related to psychiatric medication
Finding an explanation	Trusted witness to difficulties
	Psychological explanation
	Getting a diagnosis
Not being alone	Normalisation
	Safe space
	Sense of hope
	Acceptance
	Understanding
Control	Autonomy
	Communication

#### Theme one: recognising something was wrong

A theme that emerged was that participants perceived it had been difficult for them to recognise they were experiencing mental health difficulties. This appeared to result in participants ignoring early warning signs of mental health difficulties and trying to carry on until it was impossible for them to do so any longer.

Reaching a crisis point.

The participants perceived having reached a “crisis point” which meant they could not ignore the mental health difficulties they were experiencing any longer. What constituted a crisis point differed between participants and was related to factors in their environments.

P7: I can remember just being in such a state, I mean, I was seriously disturbed, so there was so many things that I felt, panic, terror, depression. I’d be, go and find a quiet spot and just break down and cry.

Difficulties experienced as physical symptoms.

The participants recalled that they first experienced physical rather than psychological symptoms.

P1: So lots of things came together at that time. My body was clearly screaming at me, I mean there were lots, all through the years actually I had lots and lots of not fully explained medical problems, which we now think were directly related to PTSD.

#### Theme two: overcoming internal stigma

One of the super-ordinate themes that emerged from the transcripts was related to how individuals perceived overcoming internal stigma related to experiencing mental health difficulties. Broadly, this fell into two areas: overcoming feelings of shame about experiencing mental health difficulties and the effect on self-esteem of being prescribed psychiatric medication.

Shame.

Participants spoke about feeling concerned that they would experience stigma, in particular, being perceived as “weak” by their peers. However, it appeared that for the majority their fears were not realised, but rather it was internal stigma they were experiencing.

Interviewer: So it sounds like you maybe had some of those fears about stigma but they weren’t realised.

P1: But actually they didn’t, they weren’t real, they didn’t, it’s not manifested itself. I think people are much more aware now of it. I think the problem was with me rather than with everybody else, it was the anticipation of stigma, maybe that says more about me than other people.

Stigma related to psychiatric medication.

Participants highlighted the link between being offered medication and internal stigma related from suffering with a mental health difficulty. They discussed their ambivalence towards medication. On the one hand, believing that medication may help them, but on the other hand, describing how taking medication meant there was something wrong with you. Medication seemed to be symbolic of having a mental illness that could no longer be ignored.

P5: I kept saying, “I’m not going on medication” but I knew I had to, I knew I needed to in the end. My mum, she’s always been on antidepressants and I thought, I always said I’d never, ever wanna be like that.

#### Theme three: finding an explanation

Participants highlighted the importance of being able to find an explanation for their difficulties. By understanding and accepting that their difficulties had a psychological component, this supported participants’ to seek help. How participants’ came to find this explanation differed greatly.

Trusted witness to difficulties.

Participants perceived the importance of having a trusted witness to their difficulties who could point out something was seriously wrong. This supported participants to accept that their difficulties were serious and that they needed to seek help.

P6: Yeah the first time round, I’ve got a very close friend in the Paras, he’s a Liaison Officer. He noticed that I was very down and I spoke differently, very slowly and I just wasn’t really interested in what he was saying and that’s not really me. I’m quite an enthusiastic outgoing person and I changed quite a lot the first time.

Psychological explanation.

Participants described how beneficial it was to be given a psychological explanation for their difficulties. This may have been because it helped them realise that their difficulties had a reason or a function.

P2: Yeah, so I have to, like when I do anything I have to sort of, I have to understand the mechanics of it, so I asked the psychiatrist how does this actually work? But if I understand the process is find it really helpful.

Getting a diagnosis.

Participants spoke about how receiving a diagnosis was a crucial step for them in their journey to seek help because it put a label on the difficulties that they were experiencing.

P8: I think I was only officially told that, you know, I think they said I had chronic PTSD and yeah it was my nurse that told me and I don’t know and then she told me, you know, she explained “These symptoms that you’re having…” And obviously there was quite a few “Is all the signs.”

P8: I was like “Jesus it must be that.” Then, I don’t know it just made me really interested, I really wanted, cause I knew what it was then and I was like “Right I can fix myself here surely.”

#### Theme four: not being alone

Another theme that emerged was related to factors that stopped participants feeling alone supported them to seek, or continue, treatment for the difficulties they were experiencing.

Normalisation.

Participants spoke about the positive experience of learning that the difficulties they were experiencing were similar to those experienced by other people.

P4: But it’s just looking into it, because when you look into it you realise, hang on, they’re talking about people going through this, this, this and this, but that’s the same as me, so you start thinking, well I’m not the only person here.

Safe space.

What appeared common across the transcripts was that having a safe space allowed participants the opportunity to take a step back and realise something was wrong; this then provided them with the motivation to seek help.

P4: I was sick on shore for two weeks. During that time it gave me time to actually rest in a secure environment because I was at home, I had my family around me. It was a secure environment. I didn’t have to look over my shoulder. And it gave me a lot of thinking time. I talked things through with my wife and thought, something’s wrong here.

Sense of hope.

Hope that things could improve was a theme that emerged in seven of the transcripts. Most of the participants recalled that hope was connected to feeling that treatment was available to help them overcome their difficulties.P1: There was part of me that was relieved, but there’s always part of me that, nobody’s harder on me than I am and, but there was also huge relief. It was, I realised that finally we may be able to do something about this.

Acceptance.

Participants spoke about the fear of not being accepted by significant people in their lives because of their mental health difficulties. However, it seems that often these fears were based on internal beliefs and not realised.

P5: I don’t even know why I was worried because I know that they wouldn’t have ever judged me but at the time that’s how I was feeling that they were gonna judge me.

Understanding.

Participants talked about how important it had been for them that other people understood the mental health difficulties they were experiencing. Participants spoke about how this had helped them not feel alone as they could share their experiences with someone who understood them.

P3: If I needed to talk to somebody about it there was always somebody that was there to talk about it. My wife really wanted to know, she’d phone me after every session to see how it had gone. And there’s a lot to take away from my sessions to share with her. And so it’s a journey we’ve been through together.

#### Theme five: control

Participants perceived that their mental health difficulties had made them feel as if they were subject to an external locus of control. In contrast, many of the participants spoke of how helpful it had been for them when engaging in help-seeking behaviour to feel an internal locus of control about their treatment options.

Autonomy.

Crucial to having a sense of control was having autonomy over their treatment plans. Tom explained how he felt supported by his line manager because they handed him control. This may be a very different experience compared to other aspects of military life, where typically service personnel have less control of their day-to-day tasks.

P3: it was a case of, well what do you want rather than them finding me something to do, what do you want to do? So I was lucky in that respect.

Communication.

Interviewed participants were worried about how they might be viewed by their friends or colleagues. They had mixed views about whether it was better to share their experiences or not.

P1 talked about how it had been a useful process for him to share his experiences with his line manager.

P1: Yeah, and once the PTSD thing had been diagnosed, actually I was given a printout of the initial session. And actually what I found the best way was actually I showed it to my boss, I said this is medically in confidence, but I said I want, I can’t really explain it but read this, and he read that bit, and from then on they couldn’t do enough, it was just.

In contrast, other participants decided that it would not be helpful to tell their colleagues.

P2: Not many people knew about it because I just walked out of this meeting and I went for a beer with an air force guy, a mate, and he just said take some time off, and that’s what I did. And of course they didn’t know that I then went and sought help. So there wasn’t some sort of big showdown, which you then had to confront going back to work.

## Discussion

The study explored which factors enabled serving members of the UK Armed Forces experiencing mental health difficulties to access care, and how they overcame common barriers to do so. To the best of the authors’ knowledge, this approach to looking at stigma and barriers to care has not been undertaken before with the UK military.

We found that all of the participants spoke about having to reach a crisis point before they sought help. What was common between the crises was individuals reaching the point where “something had to be done”; that is to say that the individual could not continue living their life as they were. Many of the participants spoke about a military culture that promotes the value of “cracking on despite a problem.” Whilst this may be advantageous in many aspects of military life, the participants spoke about how it led them to experience very serious difficulties before they would accept that they had a problem.

The majority of participants spoke about the presence of physical symptoms prior to psychological symptoms. It appears that participants expressed their psychological distress through somatic symptoms. It has previously been observed in military populations that physical health difficulties are viewed as more acceptable than mental health ones and that personnel are more likely to attend appointments for the former, rather than the latter (Rona, Jones, French, Hooper, & Wessely, 2004). This finding is mirrored when looking between cultures that have different explanations for mental illness, which can lead to either the somatic or psychological expression of symptoms. For example, Chinese people have been observed to be more likely to express symptoms of depression somatically than north-Americans (Ryder et al., 2008).

Overcoming feelings of shame about experiencing mental illness was a common theme reported by participants. Many of the participants linked accessing mental health services to their feelings of shame because this meant they had a “problem.” In addition, by accessing services it meant that their peers would also knew that they had a “problem.” These two processes map on to Corrigan’s theory of internal and external stigma (Corrigan, 2004). Participants spoke about how, over the course of engaging with services, they were able to overcome their internal stigma beliefs. For many, this process was related to realising that their negative beliefs about mental illness conflicted with the positive changes in their lives they witnessed due to seeking help. Similarly, what seemed to help the participants overcome their external stigma beliefs was the realisation that their fears of rejection from their peers were not actualised.

Three key factors that facilitated participants to engage help-seeking behaviour emerged. The first of these was being supported to develop an internal locus of control (Hiroto, 1974). Developing an internal locus of control contrasted with how the participants described their lives prior to seeking help; which for the majority, this period consisted of feeling as if there was an external locus of control. A relationship between an external locus of control and anxiety and depression has been documented by other researchers (Vuger-Kovaèiæ, Gregurek, Kovaèiæ, Vuger, & Kaleniæ, 2007). Furthermore, lower levels of anxiety and depression have been observed in individuals who report an internal, rather than an external, locus of control (Jaswel & Dewan, 1997).

The second theme that participants reported as having facilitated their accessing services was gaining a psychological understanding of their mental illness. This is supported by previous literature within civilian populations that observed having a psychological understanding predicted help-seeking behaviour (Deane, Skogstad, & Williams, 1999). Whilst the mechanisms for this relationship are unknown, from the current study it can be hypothesised that a psychological explanation was more culturally acceptable for members of the armed forces than a biological explanation, which is associated with more stigma. Indeed, many of the participants spoke about how gaining a psychological explanation helped allay their concerns about being “mad” and having something “wrong with them.”

Being well supported by their social networks was the final theme described by participants as having facilitated them to access mental health services. This finding is supported by previous research within civilian populations that documented that individuals with mental illness, who report better social support, were more likely to engage in help-seeking behaviours (Briones et al., 1990).

There are a number of limitations to this study. When interpreting these results, it is important to acknowledge that there may have been bias towards recruiting participants with lower levels of psychological distress. There was some evidence to support this in the scores reported on the measures of psychological distress. This needs to be interpreted carefully as there may have been a bias for therapists to exclude potential clients if they deemed them to be suffering from high levels of psychological distress, or only suggest potential participants who they deemed had shown significant improvement. Alternatively, it could have been that only participants who had benefitted from treatment were put forward, in which case their positive experience of treatment, may have acted to influence their recall of the factors that helped them engage in treatment by framing this decision in a potentially more positive light. It is regrettable that the authors’ do not have access to information related to stage of treatment, which may have allowed for further exploration of this. Whilst there are good clinical reasons for making these decisions, they could present limitations to the findings of the current study because individuals who have been identified as being most at risk of not being able access services are those with higher levels of psychological distress (Iversen et al., 2005a).

## Conclusions

The results of this study suggest that there are three key areas that support individuals to seek help. The first of these were factors that helped individuals recognise that they were experiencing difficulties and help them realise that these difficulties had a psychological component. The second were factors that helped an individual feel as if they were no longer alone to deal with their difficulties. For example, this included feeling accepted and supported by their social network. The final area that supported individuals to seek help was them feeling empowered to do so by having an internal locus of control. In PTSD, feelings of helplessness and powerlessness are extremely debilitating. Clinically, factors that promote an internal locus of control are very important for reducing these feelings. The participants spoke about how factors that promoted an internal locus of control helped them overcome feelings of internal stigma. It is interesting to reflect that the factors that promoted an internal locus of control could also have acted to reduce the distress caused by symptoms of PTSD by helping to tackle feelings of helplessness, isolation and powerlessness. Understanding the relevance of these three factors should help military commanders to plan effective stigma-reduction programmes.
